# The Hedgehog pathway as targetable vulnerability with 5-azacytidine in myelodysplastic syndrome and acute myeloid leukemia

**DOI:** 10.1186/s13045-015-0211-8

**Published:** 2015-10-20

**Authors:** Raoul Tibes, Aref Al-Kali, Gavin R Oliver, Devora H Delman, Nanna Hansen, Keerthi Bhagavatula, Jayaram Mohan, Fariborz Rakhshan, Thomas Wood, James M. Foran, Ruben A. Mesa, James M. Bogenberger

**Affiliations:** Division of Hematology and Medical Oncology, Mayo Clinic/Mayo Clinic Cancer Center, 13400 E. Shea Boulevard, Scottsdale, AZ 85259 USA; Mayo Clinic’s Campus in Rochester, 200 First Street SW, Rochester, MN 55905 USA; Washington University St. Louis, St. Louis, MO 63130-4899 USA; Mayo Clinic’s Campus in Florida, 4500 San Pablo Road, Jacksonville, FL 32224 USA

**Keywords:** Acute myeloid leukemia, Myelodysplastic syndrome, Hedgehog pathway/Hedgehog pathway inhibition, SMO (smoothened) inhibitor, 5-azacytidine, Erismodegib

## Abstract

**Background:**

Therapy and outcome for elderly acute myeloid leukemia (AML) patients has not improved for many years. Similarly, there remains a clinical need to improve response rates in advanced myelodysplastic syndrome (MDS) patients treated with hypomethylating agents, and few combination regimens have shown clinical benefit. We conducted a 5-azacytidine (5-Aza) RNA-interference (RNAi) sensitizer screen to identify gene targets within the commonly deleted regions (CDRs) of chromosomes 5 and 7, whose silencing enhances the activity of 5-Aza.

**Methods and results:**

An RNAi silencing screen of 270 genes from the CDRs of chromosomes 5 and 7 was performed in combination with 5-Aza treatment in four AML cell lines (TF-1, THP-1, MDS-L, and HEL). Several genes within the hedgehog pathway (HhP), specifically *SHH*, *SMO*, and *GLI3*, were identified as 5-Aza sensitizing hits. The smoothened (SMO) inhibitors LDE225 (erismodegib) and GDC0449 (vismodegib) showed moderate single-agent activity in AML cell lines. Further studies with erismodegib in combination with 5-Aza demonstrated synergistic activity with combination index (CI) values of 0.48 to 0.71 in seven AML lines. Clonogenic growth of primary patient samples was inhibited to a greater extent in the combination than with single-agent erismodegib or 5-Aza in 55 % (6 of 11) primary patient samples examined. There was no association of the 5-Aza/erismodegib sensitization potential to clinical-cytogenetic features or common myeloid mutations. Activation of the HhP, as determined by greater expression of HhP-related genes, showed less responsiveness to single-agent SMO inhibition, while synergy between both agents was similar regardless of HhP gene expression. In vitro experiments suggested that concurrent dosing showed stronger synergy than sequential dosing.

**Conclusions:**

Inhibition of the HhP with SMO inhibitors in combination with the hypomethylating agent 5-Aza demonstrates synergy in vitro and inhibits long-term repopulation capacity ex vivo in AML and MDS. A clinical trial combining 5-Aza with LDE225 (erismodegib) in MDS and AML is ongoing based on these results as well as additional publications suggesting a role for HhP signaling in myeloid disease.

**Electronic supplementary material:**

The online version of this article (doi:10.1186/s13045-015-0211-8) contains supplementary material, which is available to authorized users.

## Background

Therapeutic options for patients with acute myeloid leukemia (AML), myelodysplastic syndrome (MDS), or advanced myeloproliferative neoplasms (MPNs) are still limited, and novel molecularly targeted therapies are needed. 5-Azacytidine (5-Aza) is a hypomethylating agent (HMA) [1] commonly used as a lower intensity regimen in MDS, AML, and MPNs [2, 3]. While 5-Aza has shown clinically meaningful responses and disease control [2, 3], there remains a need to develop more effective and well-tolerated novel rational combinations [4, 5]. Deletions or monosomies of chromosomes 5 and 7 are frequent in MDS and AML and portend a worse prognosis [2, 3]. Genes on these chromosomal regions regulate tumor suppressor networks suggesting that these genes or chromosomal regions are involved in disease pathogenesis, which consequently would make them therapeutic targets either alone or with 5-Aza [6]. Thus, silencing of relevant target genes would enhance 5-Aza activity and consequently make drugs targeting such genes candidates for combination with 5-Aza. Further, a haploinsufficient therapeutic context for patients with chromosome 5/7 aberrations can be discovered, if silencing of a specific gene within these commonly deleted regions (CDRs) can be effectively inhibited and sensitizes to 5-Aza. To identify such novel target genes and molecular vulnerabilities in AML, MDS, and MPNs, we performed an RNA-interference (RNAi) screen of 270 genes located within the CDRs of chromosomes 5 and 7, in combination with 5-Aza treatment. Several genes within the Hedgehog pathway (HhP) were identified as potential sensitizers to 5-Aza. Based on these and other pre-clinical observations of a potential role of the HhP in myeloid disease as highlighted below, a clinical phase 1/1b trial of 5-Aza with the smoothened (SMO) inhibitor LDE225 (erismodegib) in AML, MDS, and MPN patients was initiated and is currently accruing patients.

The HhP is highly evolutionarily conserved and plays critical roles in embryonic development, such as regulating patterning and limb formation, and is involved in the homeostasis of many human organs and tissues including in the hematopoietic system [7]. HhP genetic alterations in the HhP are linked to the development of several human tumors such as basal cell carcinoma (BCC), medulloblastoma (MB), and rhabdomyosarcoma. Aberrant HhP signaling without evidence of genetic defects has also been linked to disease pathogenesis of many other tumors. The HhP can be activated by extracellular ligands, such as Sonic or Indian Hedgehog (SHH, IHH), that bind the transmembrane receptor Patched (PTCH) [7]. PTCH, when active, constitutively inhibits SMO and thus downstream transcription factors like GLI-1, GLI-2, GLI-3, as well as other genes involved in cell proliferation or survival (e.g., BCL-2, BCL-X_L_) [7]. Activated HhP signaling, for example, by inhibitory mutations in PTCH in BCC (i.e., the PTCH “brake” is removed) [8] or by overexpression or activation of SMO, contributes to malignant transformation via the aforementioned transcription factors and anti-apoptotic genes. Currently developed drugs, including erismodegib, mostly target/inhibit SMO as an essential intermediate gene within the HhP that is activated and mediates intracellular signaling.

A rationale for inhibiting HhP signaling in myeloid malignancies has been described in the literature based on observations that HhP signaling regulates erythroid progenitor cell proliferation and differentiation [9] and is thought to be essential for the maintenance of myeloid cancer stem cells [10]. For example, SHH activates downstream transcription factor GLI-1 in several hematological malignancies, with prevalent expression observed in AML and acute promyelocytic leukemia (APL) patients [11]. HhP genes *SHH*, *SMO*, and *GLI-1* are upregulated in chronic myeloid leukemia (CML) patients and are further elevated in blast crisis as compared to chronic-phase CML [12]. It is further hypothesized that developmental pathways such as the HhP play a role in the expansion of BCR-ABL-positive leukemic stem cells (LSCs) and may be responsible for residual disease after BCR-ABL targeted therapies [12]. Similarly, there seems to be a role for the HhP in LSCs of acute leukemias [10] and other neoplastic myeloid diseases such as MPNs [13], including polycythemia vera (PV), essential thrombocytopenia (ET), and primary myelofibrosis (PMF). Although the HhP plays a role in normal hematopoiesis and morphogenesis, the pathway is mostly silenced in normal adult tissue but re-activated in an oncogenic state. This rather selective tumor tissue expression best explains the good tolerance of SMO inhibitors in the clinic, with many patients being treated for several years [14, 15].

Preliminary clinical activity of single-agent SMO inhibitors in AML, MDS, and MPNs, including myelofibrosis (MF), has been demonstrated [16]. Herein, we report pre-clinical data of the novel combination of 5-Aza and the SMO inhibitor LDE225 in myeloid malignancies as a possible novel combination in advanced myeloid malignancies.

## Results

### RNAi screens of genes from commonly deleted regions of chromosomes 5/7 with 5-azacytidine

**Fig. 1:**
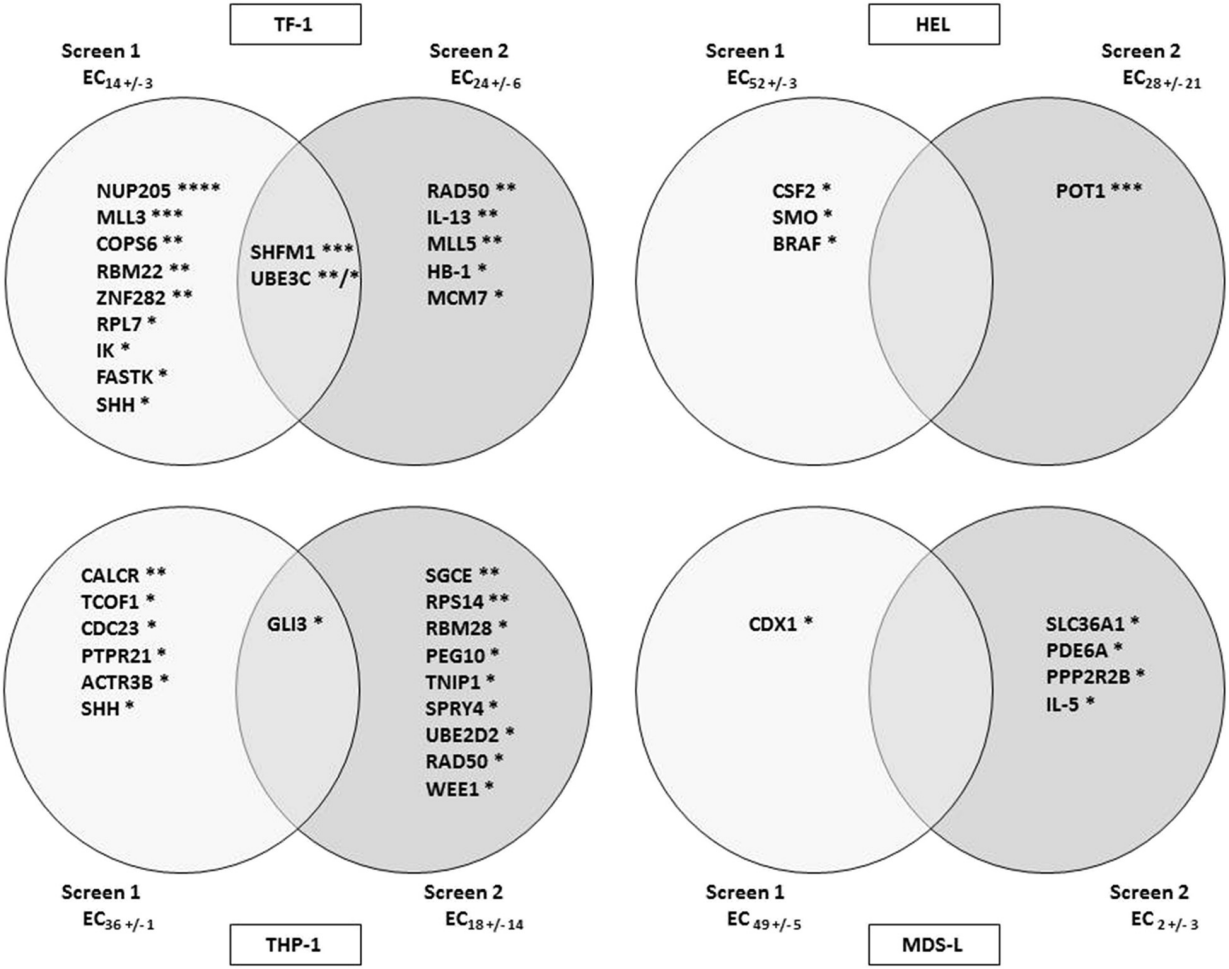
RNAi screening hits in four AML cell lines (TF-1, HEL, THP-1, MDS-L). Venn diagrams show the gene hits per screen and cell line. Hit definition/selection is described in the “Methods” section. ****3 of 3 siRNA >2 stdev; ***2 of 3 siRNA >2 stdev; **1 siRNA >2 stdev and 1 siRNA >1 stdev; *2 or 3 siRNA >1 stdev. *EC* effective concentration of 5-azacytidine at which screen was performed

To identify rational combinations with 5-Aza, a custom collection of small interfering RNA (siRNA) targeting 270 genes selected from the CDRs of chromosomes 5 and 7 was evaluated together with 5-Aza in RNAi sensitizer screens. Using four human myeloid leukemia cell lines (TF-1, HEL, THP-1, and MDS-L), several HhP genes were found to enhance 5-Aza activity. Specifically, three HhP genes emerge as sensitizers to 5-Aza when silenced by siRNA in three of four cell lines examined (Fig. 1). SHH silencing sensitized to 5-Aza in both TF-1 and THP-1 cells, while SMO and GLI-3 silencing sensitized in HEL and THP-1 cells, respectively. The RNAi screen performance was robust with high transfection efficacy (TF-1, 74–95 %; HEL, 58–86 %; THP-1, 89–96 %; MDS-L, 49–91 %), and except for THP-1, little non-specific toxicity (i.e., the non-targeting siRNA toxicity; for TF-1, 0–11 %; HEL, 0–20 %; THP-1, 34–70 %; MDS-L, 3–18 %). These performance characteristics are comparable with other RNAi screens in myeloid cells as we have reported previously [17, 18]. The effective concentration (EC) values (i.e., the percent reduction in relative cell number) for 5-Aza ranged between ~EC_5_ and EC_55_ (Fig. 1 and Additional file 1: Table S1 A–D).

### SMO inhibitors combined with 5-azacytidine in AML cells

Next, we examined the effect of two clinically developed SMO inhibitors, GDC-0449 (vismodegib), the first and only in-class approved SMO inhibitor [19], and LDE225, which is in clinical development and currently used in a combination trial with 5-Aza (clincialtrials.gov: NCT02129101). EC_50_ values for LDE225 ranged from 3.8 to 15.2 μM, and EC_50_ values for vismodegib ranged from 12 to 83 μM (Table 1). Synergy between 5-Aza and LDE225, as calculated by the Chou-Talalay method, was observed in seven molecularly heterogeneous AML cell lines with combination index (CI) values from 0.48 to 0.71 (Table 2). Importantly, synergy is observed at 5-Aza concentrations of 0.8–2.5 μM (Additional file 2: Table S2), comparable to clinically achievable 5-Aza concentrations in humans. LDE225 concentrations corresponding to optimal synergy were mostly in the range of 4–8 μM, although both higher and lower LDE225 dose outliers are observed, as low as 0.25–0.5 μM.

**Table 1:** Single-agent activity of SMO inhibitor in AML. Micromolar (μM) EC_50_ values of SMO inhibitors LDE225 (erismodegib) and GDC0449 (vismodegib) in AML cell lines

Cell line	LDE225	GDC0449
	EC_50_ [μM]	EC_50_ [μM]
MDS-L	15.2	62
TF-1 FLT3-ITD	13.6	–
UKE-1	11.9	–
HEL	11.9	83
ML-2	11.0	12
K562	10.3	32
SET-2	9.7	52
THP-1	9.4	41
OCI-AML3	9.0	38
OCI-AML2	6.9	–
MV4-11	5.4	–
M07e	5.2	–
HL-60	4.3	15
TF-1	3.8	45

**Table 2:** Synergy between 5-azacytidine and erismodegib in AML. Sensitization and synergy between LDE225 (erismodegib) and 5-azacytidine (5-Aza). EC_50_ values in micromolar (μM) and synergy presented as combination index (CI) values

Cell line	EC_50_ 5-Aza alone [μM]	EC_50_ 5-Aza + LDE225 [μM]	CI	EC_50_ LDE225 alone [μM]
TF-1	2.7	1.8	0.55	3.8
MDS-L	7.2	4.8	0.71	15.2
HL-60	1.0	0.5	0.68	4.3
MV4-11	2.2	1.1	0.68	5.4
ML-2	1.5	1.0	0.52	11
OCI-AML3	4.3	2.0	0.48	9
THP-1	21	10	0.57	10

To determine if transcript expression of relevant HhP genes correlated with single-agent activity or synergy, we assessed HhP signaling genes (e.g., *SMO*, *SHH*), HhP transcription factors (GLI-1, GLI-2, and GLI-3), and representative transcriptional target genes (e.g., anti-apoptotic *BCL-2* and BCL-XL [*BCL2L1*] and *CDK1*) for their expression in the four AML cell lines used in RNAi screening experiments (TF-1, HEL, MDS-L, and THP-1), and enriched the dataset with an additional cell line (MV4-11) for which RNA sequencing (RNAseq) data and drug treatment data was available. Overall, there was no specific distinguishing expression signature; however, general HhP activation (i.e., HEL and MDS-L) was associated with reduced sensitivity to single-agent SMO inhibition. The synergistic effects (CI values), however, were independent of HhP gene activation (Additional file 3: Figure S1), possibly suggesting that inhibiting SMO expression is more relevant in combination with 5-Aza. These gene expression experiments also indicate that different myeloid cells activate the HhP via modulation of distinct components of the HhP. For example, MDS-L displayed highest expression of IHH, PTCHD 1/2/4, and GLI1/2, whereas HEL preferentially expressed SMO and GLI2, THP-1 SHH, and SMO and GLI3. Expression levels of genes are also consistent with RNAi hits in the respective cell lines (i.e., SMO in HEL, GLI3 and SHH in THP-1). Potential differences in HhP signaling will be examined in samples from patients on trial using RNAseq as part of the biomarker analysis (clincialtrials.gov: NCT02129101).

### Hedgehog pathway inhibition in combination with 5-azacytidine in clonogenic assays

**Fig. 2:**
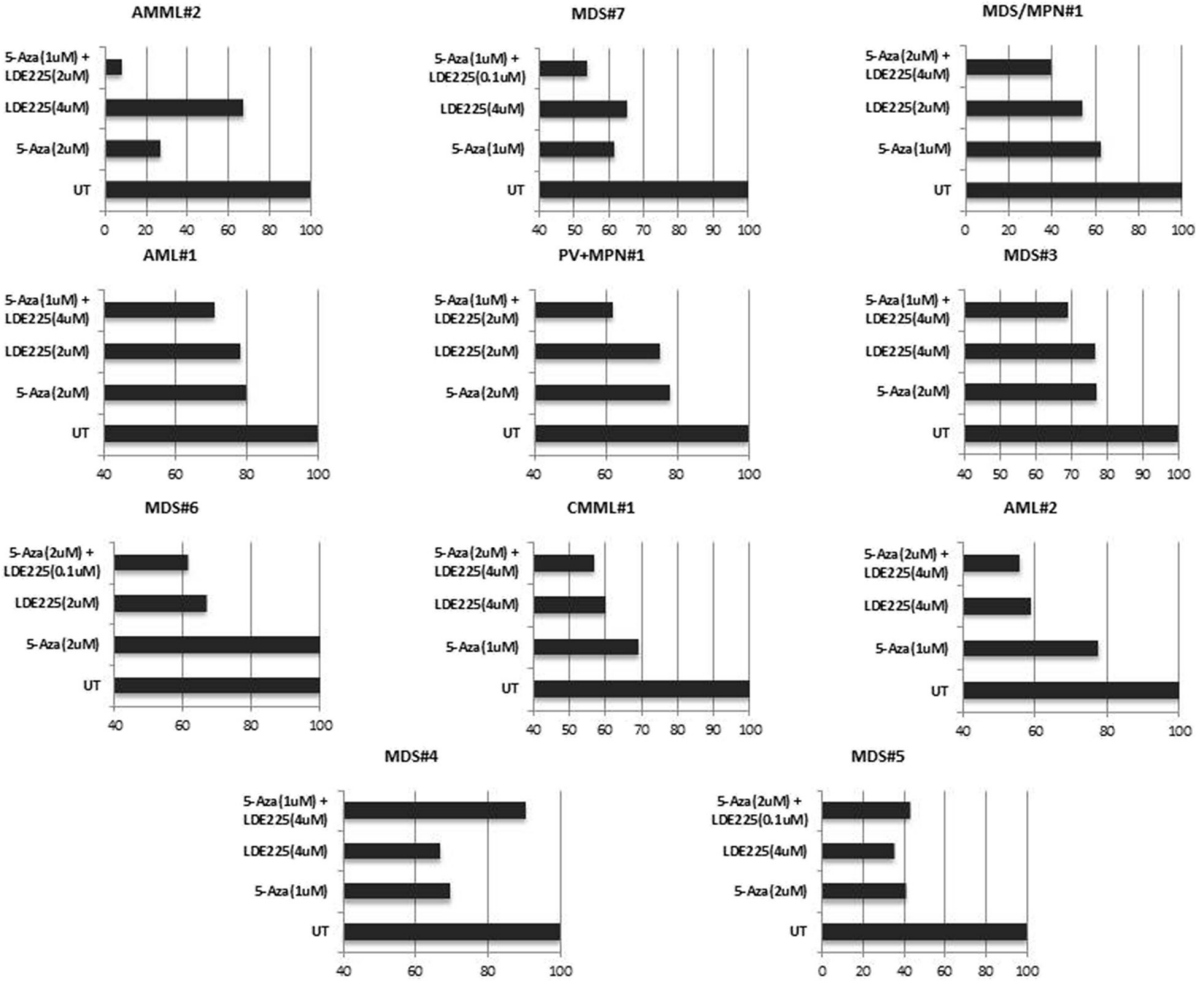
Clonogenic growth inhibition of primary MDS and AML specimens. LDE225 (erismodegib) and 5-azacytidine in primary patient samples. *UT* untreated. Dose of 5-Aza and LDE225 given in micromolar at *y*-axis. Percent growth at *x*-axis

Strong sensitization with greatly reduced colony count is observed with combined LDE225 and 5-Aza as compared to either single agent in several primary MDS and AML samples in clonogenic assays (Fig. 2, i.e., AMML#2, MDS#7, PV+MPN#1). However, some samples showed a neutral response to the combination compared to one or both single agent (i.e., AML#1, AML#2, MDS#3), while a few samples do not show added benefit over the combination, and may even exhibit slight antagonism with combination treatment compared to either single agent (i.e., MDS#4, MDS#5). The greatest degree of sensitization is seen at 2–4 μM of LDE225, at a 5-Aza dose kept constant at 1 and 2 μM to reflect clinically achievable concentrations of 5-Aza. Interestingly, one sample (MDS#3) assessed at the time of MDS diagnosis showed little benefit from the combination, yet a sample from this same patient drawn and assayed at the time of progression to AML (AML#2) showed increased benefit from both single-agent LDE225 and the LDE225/5-Aza combination. The clinical data and molecular characteristics of patient samples examined in ex vivo clonogenic assays are shown in Table 3. There was no correlation in this dataset to any obvious clinical molecular-cytogenetic characteristics or to targeted sequencing of mutations in myeloid-associated genes performed on a number of samples.

**Table 3:** Clinical and molecular patient characteristics

Patient	Age	Gender	Disease	Counts				Treatment status	Cytogenetics		(Standard) molecular tests	Targeted sequencing
				WBC	% Blasts	Hb (g/dL)	Platelets		CG	FISH		
MDS#1	75	M	MDS/MPN	100	0–1	10–11	15	Naïve (except prior HU)	46 xy	Not done	JAK2 pos. MPL neg.	Not done
AMML#2	65	M	CMML->AMML	6	17–21	8.8	107	Naïve	46 xy	Normal	JAK/MPL/BCR-ABL all neg.	Not done
MDS#3^a^	65	M	RCMD/RAEB-1	1.7	4	7–8	56	Lenalidomide, azacitidine decitabine (off >4 months)	del(5) (q13q33), del(20) (q11.2q13.3)	5q-(85 %), del20q (64 %)	None	NF1, TET2, RUNX1, BCOR, EZH2, SF3B1
AML#2^a^	65	M	MDS->AML	2.1	52	8.4	25	Lenalidomide, azacitidine decitabine (off >8 months at AML progression)	del(5) (q13q33), del(20) (q11.2q13.3)	5q-(85 %), 20q- (11.5 %)	None	NF1, TET2, RUNX1, BCOR, EZH2, SF3B1
MDS#4	70	M	RCMD	1.7	1	11.3	80	2-CDA for HCL 8 years prior	del 20q (q11.2q13.3)	20q- (38.5 %) +8 (7.5 %)	Not done	Not done
MDS#5	73	M	MDS->AML	0.8	5	9.7	11	Progressed after CR azacitidine, minimal/no response to decitabine	46 xy	Normal	FLT3 neg.	NRAS, NF-1, MLL-PTD, DNMT3A, BCOR, U2AF1
MDS#6	73	M	RAEB-2->AML	2.7	12	10.3	11	No response to oral azacitidine	46 xy	Normal	FLT3 neg. NPM1 neg.	No mutation detected
MDS#7	77	M	RCMD	12.5	1	435	435	2-CDA for MM 3 years prior	Complex [del1p, 5q22del translocation 6; 7, +8]	5q- (28.5 %) +8 (20.5 %) +3q21, +3q26.2 (both 54 %)	Not done	Not done
AML#1	72	M	AML (de novo)	–	–	–	–	Naïve	del(9) (q13q22) -y	Normal	FLT neg. NPM pos.	Not done
CMML#1	84	F	CMML-1	20	1	9	169	Azacitidine + SAHA (off 4 months)	46 xx	Normal	None	TET2, NRAS, MLL-PTD, RUNX1, ASXL1, EZH2, WT1

^a^Same patient analyzed at time of MDS and at later stage after AML evolution

### Sequencing schedules of combined 5-azacytidine and SMO inhibitors and specificity to HMA

As the optimal sequence of SMO inhibition with 5-Aza is not determined, drug sequencing experiments using high, middle, and low LDE225 concentrations were performed on standard doses of 5-Aza in vitro. As shown in Table 4, concurrent treatment at higher doses of 8 and 32 μM LDE225 showed greatest sensitization by fold-shift in EC_50_ values of 5-Aza, while sequential dosing showed a trend towards antagonism, at least in vitro.

**Table 4:** Sequential versus concurrent 5-azacytidine/LDE225 treatment

Cell line	LDE225 dose and sequence of drug dosing								
	32 μM LDE225			8 μM LDE225			2 μM LDE225		
	5-Aza + LDE225	LDE225→5-Aza	5-Aza→LDE225	5-Aza + LDE225	LDE225→5-Aza	5-Aza→LDE225	5-Aza + LDE225	LDE225→5-Aza	5-Aza→LDE225
TF-1	1.3	−1.5	1	1.2	−1.6	−1.2	1.3	−1.1	−1.1
OCI-AML3	2.6	−2	−1.5	2	−1.8	−1.7	1.3	−1.3	−1.2
MDS-L	1.5	−1.6	−1.2	1.5	−1.1	−1.2	1.1	−1.2	−1.2
THP-1	2	−1	−1.1	2	−1.5	−1.3	1.1	−1.3	−1.2

Fold-shift of EC_50_ values at sequential (5-Aza first followed by LDE-225: 5-Aza→LDE225; LDE225 first followed by 5-Aza: LDE225→5-Aza) or concurrent (5-Aza + LDE225) treatment in four AML cell lines. Fold-shift is calculated by comparing the 5-Aza EC50 value in the combination of 5-Aza + LDE225 to the EC50 value of 5-Aza alone

A comparison of the synergistic potential of erismodegib with 5-Aza versus cytarabine (Ara-C), the most commonly used AML cytotoxic drug, shows that while sensitization is observed with 5-Aza, antagonism is observed when combined with Ara-C, demonstrated by a rightward curve shift to a higher EC_50_ value (Additional file 4: Figure S2).

## Conclusions

RNAi screening of AML and MDS relevant genes located on chromosomes 5 and 7 in combination with 5-Aza yielded potential targetable molecular vulnerabilities. Several of the 5-Aza sensitizing gene hits are situated within the HhP. Experiments with SMO inhibitors in vitro and ex vivo pharmacologically validate the idea that the HhP could serve as a therapeutic target with HMAs in myeloid malignancies. This is of immediate translational relevance as several well-tolerated SMO inhibitors are clinically developed. Trials of SMO inhibitors and HMAs, either 5-Aza or decitabine (DAC), are currently ongoing in patients with AML, MDS, and MPNs. To our knowledge, this is the first report showing synergy between an SMO inhibitor and a HMA in primary AML and MDS samples, as well as in AML cell lines.

The HhP is highly complex and difficult to examine in vitro and even in vivo. Thus, detailed molecular analyses of potential underlying mechanisms for the sensitization effects were not performed in this study. Because clinical development is proceeding so rapidly, future biology will be best explored using actual samples from patients treated on study. To that end, we are prospectively collecting sequential samples from patients on an ongoing trial combining 5-Aza with erismodegib (NCT02129101).

Foregoing an investigation of the complex HhP biology pre-clinically, we will focus efforts on exploring the biology of HhP and SMO inhibitors in situ with actual samples from patients on trial. We will perform global RNA sequencing and complementary genomic assays on serially collected samples to assess baseline and transcriptional changes associated with clinical response to SMO inhibition in AML and MDS. We prefer this in situ approach, as the ultimate proof of effectiveness for a novel combination can only be shown in the clinic, and novel combinations are urgently needed for advanced MDS and elderly AML patients. Consequently, the goal of the present study was to provide preliminary evidence of the sensitization and interaction between 5-Aza and SMO inhibition in primary malignant myeloid cells ex vivo, which has not been reported previously. Furthermore, RNAi screens have inherent biases for both false positives and negatives (i.e., hits derived from off-target activity and genes missed by insufficient silencing); thus, there are possibly additional target genes on the CDRs of chromosomes 5 and 7 that may sensitize to HMAs. Despite these limitations, RNAi screens can serve as a valuable assay to identify leads that can guide further pre-clinical validation and translation into the clinic.

Several observations regarding the sensitization between 5-Aza and SMO inhibition are worth noting. First, sensitization is at least partially independent of surrounding stroma cells and cellular structures indicating that HhP inhibition with SMO inhibitors in myeloid cells is at least partially cell autonomous (i.e., autocrine) or paracrine between malignant myeloid cells. Second, not all specimens showed sensitization in ex vivo assays. Sensitization was observed in approximately 30 to 50 % of samples making biomarker studies with actual patient samples even more important once outcome and clinical response assessments are available. Within the clinical characteristics of the patient samples examined ex vivo in this study, there was no apparent feature differentiating responding (sensitized) samples, based on cytogenetics, disease subtype, or targeted mutation profiling, from non-responding patient samples (Table 3). HhP gene activation appears to associate with single-agent sensitivity to SMO inhibitors (Additional file 3: Figure S1), whereas there was no apparent association with combination synergy. Limited pre-clinical in vitro data showed that concurrent treatment of erismodegib together with 5-Aza may be effective, which has informed trial design by adding a treatment arm with concurrent dosing of 5-Aza and SMO inhibitors. Concurrent treatment may allow SMO inhibitors to be further escalated to a dose that may otherwise not be tolerated if given continuously. We are exploring this concept in the ongoing trial (R. Tibes, personal communication).

In conclusion, targeting the HhP by inhibiting SMO, in combination with the HMA 5-Aza, shows sensitization in some, but not all, primary AML, MDS, and MPN patient samples. The mechanism(s) of synergy remain uncertain and require further investigation in future studies. Given the overall good clinical tolerance of SMO inhibitors, the activity of 5-Aza in MDS and AML, and pre-clinical studies presented herein, the rational combination of erismodegib and 5-Aza is being examined in an ongoing clinical trial.

## Methods

### Cell lines, primary sample isolation, culture conditions, and reagents

Human acute myeloid leukemia cell lines TF-1, THP-1, HEL, and MDS-L [20] and primary patient samples were cultured at 37 °C under 5 % CO_2_ atmosphere in culture medium consisting of RPMI-1640 supplemented with 10 % fetal bovine serum, 2-mM l-glutamine, 100 IU/mL penicillin, and 100 μg/mL streptomycin. Primary samples were collected from patients with informed consent under Mayo Clinic IRB-approved research protocols and handled according to Good Clinical Practice. Primary cells were Ficoll-gradient separated and used as outlined and previously [18, 21]. All reagents for cell culture were obtained from Invitrogen (Carlsbad, CA, USA). Culture media for TF-1 and MDS-L were supplemented with 10 ng/mL GM-CSF or IL-3 (Stem Cell Technologies, Vancouver, BC, CA), respectively. 5-Aza was obtained from Sigma-Aldrich (St. Louis, MI, USA), GDC0449 from SeleckChem (Houston, TX, USA), and LDE225 was obtained from Novartis as well as purchased from SeleckChem.

### RNA-interference screens

A custom siRNA library targeting 270 genes derived from the commonly deleted regions (CDRs) of chromosomes 5/7 with 3× siRNA sequences per gene was assembled (Qiagen, Valencia, CA, USA). Genes were silenced by delivery of siRNA with cationic lipid-based transfection reagents for 48 h followed by 5-Aza treatment or treatment with culture medium for siRNA-only control plates, for an additional 48 h, after which relative cell number/cell viability was determined using CellTiter-Glo (Promega, Madison, WI, USA). All RNAi screen plates contained non-silencing siRNA and universal lethal siRNA controls. Hits were selected as >2 standard deviation changes in viability from the median log2 value of the ratio [(siRNA + 5-Aza) / (siRNA only)]. RNAi screens were performed in duplicate.

### Drug-dose response assays and CalcuSyn analysis

Combination drug-dose response assays were performed similar to previous descriptions [18, 21]. In brief, 384-well plates were used to assess nine doses of 5-Aza diluted threefold serially and six doses of LDE225 diluted two- to fourfold serially, yielding possible 54 dose combination readouts, each combination having quadruplicate data points. Relative cell number/viability was determined at 96 h using CellTiter-Glo (Promega) for all drug-dose response assays. For experiments analyzing sequential drug dosing, the second drug was administered 48 h after the first drug. Prism Version 5.03 (Prism Software Corporation, Irvine, CA, USA) was used for calculating 5-Aza EC_50_ values. CI values were determined using CalcuSyn Version 2.1 (Biosoft, Cambridge, UK) as developed by Chow and Talalay [22].

### RNA sequencing

#### Sample preparation and sequencing

RNA libraries were prepared according to the manufacturer’s instructions for the TruSeq RNA Sample Prep Kit v2 (Illumina, San Diego, CA). The liquid handling Eppendorf (Hamburg, GER) EpMotion 5075 robot was employed for TruSeq library construction. All AMPure bead cleanup, messenger RNA (mRNA) isolation, end repair, and A-tailing reactions were completed on the 5075 robot. Reverse transcription and adaptor ligation steps were performed manually. Briefly, poly-A mRNA was purified from total RNA using oligo dT magnetic beads. The purified mRNA was fragmented at 95 °C for 8 min, eluted from the beads, and primed for first-strand complementary DNA (cDNA) synthesis. The RNA fragments were then copied into first-strand cDNA using SuperScript III reverse transcriptase and random primers (Invitrogen, Carlsbad, CA). Next, second-strand cDNA synthesis was performed using DNA polymerase I and RNase H. The double-stranded cDNA was purified using a single AMPure XP bead (Agencourt, Danvers, MA) cleanup step. The cDNA ends were repaired and phosphorylated using Klenow, T4 polymerase, and T4 polynucleotide kinase followed by a single AMPure XP bead cleanup. The blunt-ended cDNAs were modified to include a single 3′ adenylate (A) residue using Klenow exo- (3′ to 5′ exo minus). Paired-end DNA adaptors (Illumina) with a single “T” base overhang at the 3′ end were immediately ligated to the “A tailed” cDNA population. Unique indexes, included in the standard TruSeq Kits (12-Set A and 12-Set B) were incorporated at the adaptor ligation step for multiplex sample loading on the flow cells. The resulting constructs were purified by two consecutive AMPure XP bead cleanup steps. The adapter-modified DNA fragments were enriched by 12 cycles of PCR using primers included in the Illumina Sample Prep Kit. The concentration and size distribution of the libraries were determined on an Agilent Bioanalyzer DNA 1000 chip (Santa Clara, CA). A final quantification, using Qubit fluorometry (Invitrogen, Carlsbad, CA), was done to confirm sample concentration.

Two RNAseq libraries per lane were loaded onto paired-end flow cells at concentrations of 8–10 pM to generate cluster densities of 700,000–800,000/mm^2^ following Illumina’s standard protocol using either the Illumina cBot or HiSeq 2500 and TruSeq Rapid Paired-End cluster kit version 1.

The flow cells were sequenced as 100 × 2 paired-end reads on an Illumina HiSeq 2500 using TruSeq Rapid SBS sequencing kit version 1 and HCS version 2.0.12.0 data collection software. Base-calling is performed using Illumina’s RTA version 1.17.21.3.

#### RNA analysis

FastQC (v 0.10) and RSeQC (1) were used to monitor read quality. RNA analysis was performed using an internally developed pipeline called MAP-RSeq (2). Briefly, reads were aligned to the human genome (hg19) and transcriptome using Tophat2 (3) running Bowtie (v1) (4). Gene and exon level read counts were generated using HtSeq (5) and BedTools (6), respectively, and normalized using RPKM normalization. Data analysis was conducted using the R package and Qiagen’s Ingenuity® Pathway Analysis (IPA®, Qiagen Redwood City, www.qiagen.com/ingenuity).

### Clonogenic assays

Briefly, as previously described [4, 21], cells were suspended in Methocult H4434 Classic (Stem Cell Technologies) and dosed with 5-Aza and/or LDE225. Duplicate 35-mm dishes were plated for each experimental treatment and allowed to incubate for 11–14 days at 37 °C under 5 % CO_2_ atmosphere before counting total colonies on treatment blinded dishes. Treatment effect on colony growth was determined by dividing the average colony count of both dishes for each treatment by the average colony count of both untreated control dishes.

## Additional files

Additional file 1: Table S1. A–D. Additional file 2: Table S2. Detailed synergy between LDE225 and 5-Azacytidine in AML Cell Line. Additional file 3: Figure S1. Top: Heatmap of 21 HhP genes clustered by cell line. Bottom: Table with single-agent EC_50_ in *μM* and synergy potential expressed as Combination Index (C.I.) values. Additional file 4: Figure S2. 5-Azacytidine (5-Aza) showed curve shift to lower EC_50_ values, whereas cytarabine (Ara-C) showed the opposite trend in viability assays.

## Abbreviations

5-Aza: 5-azacytidine
AML: acute myeloid leukemia
APL: acute promyelocytic leukemia
BCC: basal cell carcinoma
CDRs: commonly deleted regions
CI: combination indices
CML: chronic myeloid leukemia
EC: effective concentration
ET: essential thrombocytopenia
HhP: Hedgehog pathway
HMA: hypomethylating agent
IHH: Indian Hedgehog
LSCs: leukemic stem cells
MB: medulloblastoma
MDS: myelodysplastic syndrome
MF: myelofibrosis
MPNs: myeloproliferative neoplasms
PMF: primary myelofibrosis
PTCH: transmembrane receptor Patched
PV: polycythemia vera
RNAi: RNA-interference
RNAseq: RNA sequencing
SHH: Sonic Hedgehog
SMO: smoothened

## References

[1] Gutierrez SE, Romero-Oliva FA (2013). Epigenetic changes: a common theme in acute myelogenous leukemogenesis. J Hematol Oncol.

[2] Pleyer L, Stauder R, Burgstaller S, Schreder M, Tinchon C, Pfeilstocker M (2013). Azacitidine in patients with WHO-defined AML—results of 155 patients from the Austrian Azacitidine Registry of the AGMT-Study Group. J Hematol Oncol.

[3] van der Helm LH, Scheepers ER, Veeger NJ, Daenen SM, Mulder AB, van den Berg E (2013). Azacitidine might be beneficial in a subgroup of older AML patients compared to intensive chemotherapy: a single centre retrospective study of 227 consecutive patients. J Hematol Oncol.

[4] Bogenberger JM, Delman D, Hansen N, Valdez R, Fauble V, Mesa RA (2015). Ex vivo activity of BCL-2 family inhibitors ABT-199 and ABT-737 combined with 5-azacytidine in myeloid malignancies. Leuk Lymphoma.

[5] Carter BZ, Mak PY, Mak DH, Shi Y, Qiu Y, Bogenberger JM (2014). Synergistic targeting of AML stem/progenitor cells with IAP antagonist birinapant and demethylating agents. J Natl Cancer Inst.

[6] Eisenmann KM, Dykema KJ, Matheson SF, Kent NF, DeWard AD, West RA (2009). 5q- myelodysplastic syndromes: chromosome 5q genes direct a tumor-suppression network sensing actin dynamics. Oncogene.

[7] Tibes R, Mesa RA (2014). Targeting hedgehog signaling in myelofibrosis and other hematologic malignancies. J Hematol Oncol.

[8] Jimeno A, Weiss GJ, Miller WH, Gettinger S, Eigl BJ, Chang AL (2013). Phase I study of the Hedgehog pathway inhibitor IPI-926 in adult patients with solid tumors. Clin Cancer Res.

[9] Detmer K, Thompson AJ, Garner RE, Walker AN, Gaffield W, Dannawi H (2005). Hedgehog signaling and cell cycle control in differentiating erythroid progenitors. Blood Cells Mol Dis.

[10] Zhao C, Chen A, Jamieson CH, Fereshteh M, Abrahamsson A, Blum J (2009). Hedgehog signalling is essential for maintenance of cancer stem cells in myeloid leukaemia. Nature.

[11] Bai LY, Chiu CF, Lin CW, Hsu NY, Lin CL, Lo WJ (2008). Differential expression of Sonic hedgehog and Gli1 in hematological malignancies. Leukemia.

[12] Long B, Zhu H, Zhu C, Liu T, Meng W (2011). Activation of the Hedgehog pathway in chronic myelogeneous leukemia patients. J Exp Clin Cancer Res.

[13] Zingariello M, Martelli F, Ciaffoni F, Masiello F, Ghinassi B, D’Amore E (2013). Characterization of the TGF-beta1 signaling abnormalities in the Gata1low mouse model of myelofibrosis. Blood.

[14] LoRusso PM, Rudin CM, Reddy JC, Tibes R, Weiss GJ, Borad MJ (2011). Phase I trial of hedgehog pathway inhibitor vismodegib (GDC-0449) in patients with refractory, locally advanced or metastatic solid tumors. Clin Cancer Res.

[15] Weiss GJ, Tibes R, Blaydorn L, Jameson G, Downhour M, White E (2011). Long-term safety, tolerability, and efficacy of vismodegib in two patients with metastatic basal cell carcinoma and basal cell nevus syndrome. Dermatol Rep.

[16] Jamieson C, Cortes JE, Oehler V, Baccarani M, Kantarjian HM, Papayannidis C (2011). Phase 1 dose-escalation study of PF-04449913, an oral Hedgehog (Hh) inhibitor, in patients with select hematologic malignancies. ASH Ann Meet Abstr.

[17] Chaudhuri L, Vincelette ND, Koh BD, Naylor RM, Flatten KS, Peterson KL (2014). CHK1 and WEE1 inhibition combine synergistically to enhance therapeutic efficacy in acute myeloid leukemia ex vivo. Haematologica.

[18] Tibes R, Bogenberger JM, Chaudhuri L, Hagelstrom RT, Chow D, Buechel ME (2012). RNAi screening of the kinome with cytarabine in leukemias. Blood.

[19] Von Hoff DD, LoRusso PM, Rudin CM, Reddy JC, Yauch RL, Tibes R (2009). Inhibition of the hedgehog pathway in advanced basal-cell carcinoma. N Engl J Med.

[20] Nakamura S, Ohnishi K, Yoshida H, Shinjo K, Takeshita A, Tohyama K (2000). Retrovirus-mediated gene transfer of granulocyte colony-stimulating factor receptor (G-CSFR) cDNA into MDS cells and induction of their differentiation by G-CSF. Cytokines Cell Mol Ther.

[21] Bogenberger JM, Kornblau SM, Pierceall WE, Lena R, Chow D, Shi CX (2014). BCL-2 family proteins as 5-azacytidine-sensitizing targets and determinants of response in myeloid malignancies. Leukemia.

[22] Reynolds CP, Maurer BJ (2005). Evaluating response to antineoplastic drug combinations in tissue culture models. Methods Mol Med.

