# Supplementary material for: The Hedgehog pathway as targetable vulnerability with 5-azacytidine in myelodysplastic syndrome and acute myeloid leukemia
Source: J Hematol Oncol. 2015 Oct 20;8:114. doi: 10.1186/s13045-015-0211-8 (PMC4615363; doi:10.1186/s13045-015-0211-8)
Supplement: Additional file 1: Table S1. — A–D. [file 13045_2015_211_MOESM1_ESM.docx]

**Table S1 A-D**

| **A)** | **TF-1** | **Screen 1** | | | **Screen 2** | | |
| --- | --- | --- | --- | --- | --- | --- | --- |
|  |  | ***Plate 1-1***  ***Chr 5*** | ***Plate 1-2***  ***Chr 7*** | ***Plate 2-2***  ***Chr 7*** | ***Plate 1-1***  ***Chr 5*** | ***Plate 1-2***  ***Chr 7*** | ***Plate 2-2***  ***Chr 7*** |
|  | % Transfection Efficiency | 83 | 74 | 87 | 95 | 94 | 95 |
|  | % Non-specific Toxicity | 1 | 0 | 1 | 1 | 6 | 11 |
|  | 5-Aza EC Value | 15 | 10 | 16 | 31 | 19 | 21 |
| **B)** | **THP-1** | **Screen 1** | | | **Screen 2** | | |
|  |  | ***Plate 1-1***  ***Chr 5*** | ***Plate 1-2***  ***Chr 7*** | ***Plate 2-2***  ***Chr 7*** | ***Plate 1-1***  ***Chr 5*** | ***Plate 1-2***  ***Chr 7*** | ***Plate 2-2***  ***Chr 7*** |
|  | % Transfection Efficiency | 96 | 92 | 96 | 89 | 94 | 92 |
|  | % Non-specific Toxicity | 70 | 44 | 71 | 34 | 57 | 49 |
|  | 5-Aza EC Value | 37 | 35 | 35 | 10 | 10 | 35 |
| **C)** | **HEL** | **Screen 1** | | | **Screen 2** | | |
|  |  | ***Plate 1-1***  ***Chr 5*** | ***Plate 1-2***  ***Chr 7*** | ***Plate 2-2***  ***Chr 7*** | ***Plate 1-1***  ***Chr 5*** | ***Plate 1-2***  ***Chr 7*** | ***Plate 2-2***  ***Chr 7*** |
|  | % Transfection Efficiency | 84 | 58 | 77 | 74 | 86 | 83 |
|  | % Non-specific Toxicity | 16 | 0 | 16 | 9 | 14 | 20 |
|  | 5-Aza EC Value | 55 | 52 | 50 | 40 | 4 | 39 |
| **D)** | **MDS-L** | **Screen 1** | | | **Screen 2** | | |
|  |  | ***Plate 1-1***  ***Chr 5*** | ***Plate 1-2***  ***Chr 7*** | ***Plate 2-2***  ***Chr 7*** | ***Plate 1-1***  ***Chr 5*** | ***Plate 1-2***  ***Chr 7*** | ***Plate 2-2***  ***Chr 7*** |
|  | % Transfection Efficiency | 77 | 91 | 77 | 49 | 57 | 51 |
|  | % Non-specific Toxicity | 9 | 3 | 10 | 12 | 12 | 18 |
|  | 5-Aza EC Value | 53 | 43 | 50 | 0 | 1 | 5 |
