# Supplementary material for: The Hedgehog pathway as targetable vulnerability with 5-azacytidine in myelodysplastic syndrome and acute myeloid leukemia
Source: J Hematol Oncol. 2015 Oct 20;8:114. doi: 10.1186/s13045-015-0211-8 (PMC4615363; doi:10.1186/s13045-015-0211-8)
Supplement: Additional file 2: Table S2. — Detailed synergy between LDE225 and 5-Azacytidine in AML Cell Line. [file 13045_2015_211_MOESM2_ESM.docx]

**Table S2**: **Detailed synergy between LDE225 and 5-Azacytidine in AML Cell Line**

Various concentrations of 5-Azacytidine (5-Aza) and LDE225 in AML cell lines. Synergy is presented as Combination Index (C.I.) values, with the *µM* dose of LDE225 listed below each C.I. value.

| **Cell Line** | **C.I.**  **5-Aza**  **0.09 µM** | **C.I.**  **5-Aza**  **0.27 µM** | **C.I.**  **5-Aza**  **0.82 µM** | **C.I.**  **5-Aza**  **2.5 µM** | **C.I.**  **5-Aza**  **7.4 µM** | **C.I.**  **5-Aza**  **22 µM** | **C.I.**  **5-Aza**  **66/200 µM** |
| --- | --- | --- | --- | --- | --- | --- | --- |
| **TF-1** | - | 0.42  (0.25 µM) | 0.71  (8 µM) | 0.75  (8 µM) | 0.67  (8 µM) | 0.54  (8 µM) | 0.32  (4 µM) |
| **MDS-L** | - | - | - | 0.76  (16 µM) | - | 0.6  (2 µM) | 0.6  (2 µM) |
| **HL-60** | 0.65  (1 µM) | 0.76  (8 µM) | 0.68  (8 µM) | 0.54  (8 µM) | 0.51  (8 µM) | 0.51  (1 µM) | - |
| **MV4-11** | 0.61  (2 µM) | 0.79  (32 µM) | - | 0.67  (32 µM) | 0.29  (16 µM) | 0.36  (1 µM) | - |
| **ML-2** | 0.67  (16 µM) | 0.6  (16 µM) | 0.49  (4 µM) | 0.68  (32 µM) | 0.53  (32 µM) | 0.42  (32 µM) | - |
| **OCI-AML3** | 0.75  (2 µM) | 0.67  (2 µM) | 0.62  (2 µM) | 0.47  (8 µM) | 0.19  (8 µM) | - | - |
| **THP-1** | - | - | 0.55  (0.5 µM) | 0.64  (32 µM) | 0.57  (32 µM) | 0.5  (32 µM) | 0.16  (32 µM) |
