# Supplementary figures and images for: The Hedgehog pathway as targetable vulnerability with 5-azacytidine in myelodysplastic syndrome and acute myeloid leukemia
Source: J Hematol Oncol. 2015 Oct 20;8:114. doi: 10.1186/s13045-015-0211-8 (PMC4615363; doi:10.1186/s13045-015-0211-8)

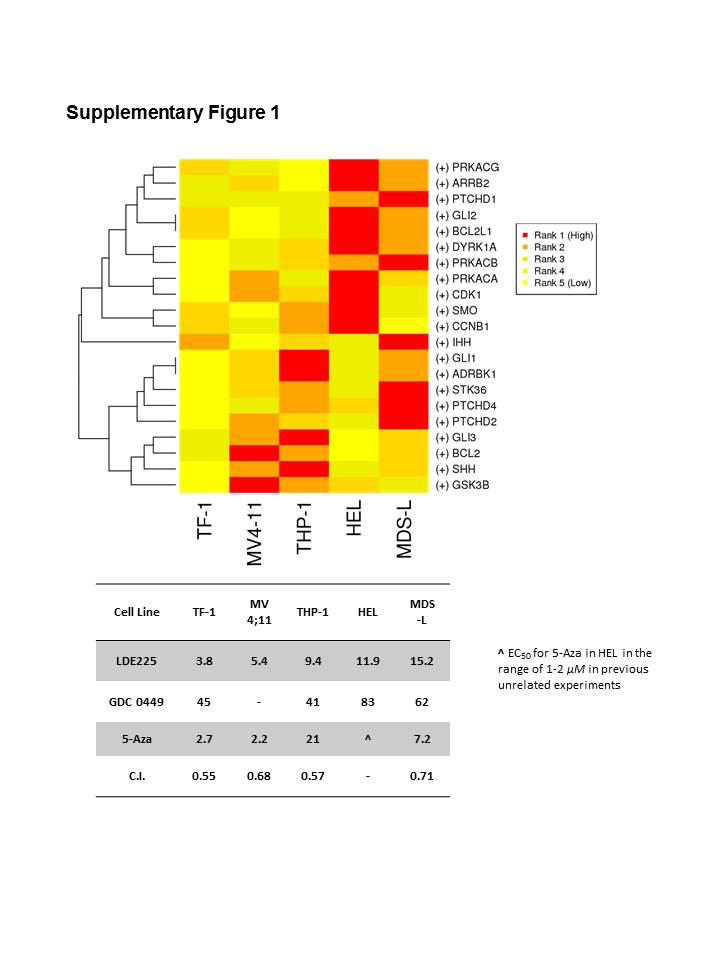

Supplement: Additional file 3: Figure S1. — Top: Heatmap of 21 HhP genes clustered by cell line. Bottom: Table with single-agent EC50 in μM and synergy potential expressed as Combination Index (C.I.) values. [file 13045_2015_211_MOESM3_ESM.jpg]

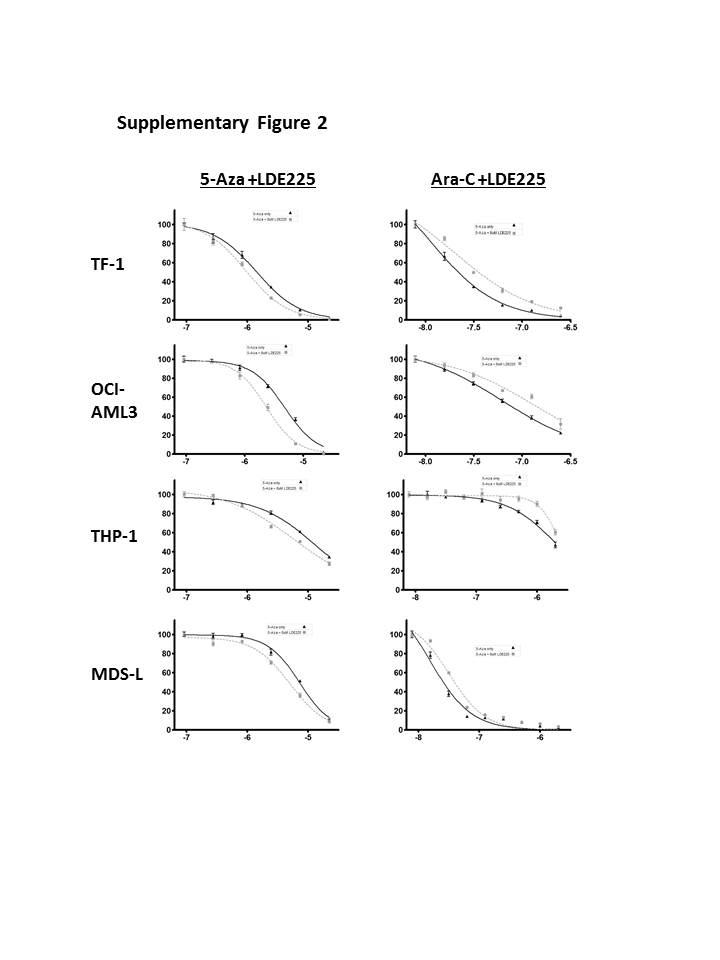

Supplement: Additional file 4: Figure S2. — 5-Azacytidine (5-Aza) showed curve shift to lower EC50 values, whereas cytarabine (Ara-C) showed the opposite trend in viability assays. [file 13045_2015_211_MOESM4_ESM.jpg]
